# Knowledge of Rare Respiratory Diseases among Paediatricians and Medical School Students

**DOI:** 10.3390/jcm9030869

**Published:** 2020-03-22

**Authors:** María Ángeles Requena-Fernández, Francisco Dasí, Silvia Castillo, Rafael Barajas-Cenobi, María Mercedes Navarro-García, Amparo Escribano

**Affiliations:** 1 Paediatrics Unit, Hellín Hospital, 02400 Albacete, Spain; ma.reke@hotmail.com; 2 Fundación Investigación Hospital Clínico Universitario de Valencia/Instituto de Investigación Sanitaria INCLIVA, 46010 Valencia, Spain; castillo_sil@gva.es (S.C.); rbarajas@incliva.es (R.B.-C.); mer_navarro2002@yahoo.es (M.M.N.-G.); aescribano@separ.es (A.E.); 3 Department of Physiology, School of Medicine, University of Valencia, 46010 Valencia, Spain; 4 Paediatrics Pulmonology Unit, Hospital Clínico Universitario Valencia, 46010 Valencia, Spain; 5 Department of Paediatrics, Obstetrics and Gynaecology, School of Medicine, University of Valencia, 46010 Valencia, Spain

**Keywords:** alpha-1 antitrypsin deficiency, primary ciliary dyskinesia, rare respiratory diseases

## Abstract

Alpha-1-antitrypsin deficiency (AATD) and primary ciliary dyskinesia (PCD) are underdiagnosed rare diseases showing a median diagnostic delay of five to ten years, which has negative effects on patient prognosis. Lack of awareness and education among healthcare professionals involved in the management of these patients have been suggested as possible causes. Our aim was to assess knowledge of these diseases among paediatricians and medical school students to determine which knowledge areas are most deficient. A survey was designed with questions testing fundamental aspects of the diagnosis and treatment of AATD and PCD. A score equal to or greater than 50% of the maximum score was set as the level necessary to ensure a good knowledge of both diseases. Our results indicate a profound lack of knowledge of rare respiratory diseases among paediatric professionals and medical students, suggesting that it is necessary to increase rare respiratory diseases training among all physicians responsible for suspecting and diagnosing them; this will allow early diagnosis and the setup of preventive measures and appropriate early-stage treatment. The first step in closing this knowledge gap could be to include relevant material in the medical syllabus.

## 1. Introduction

Alpha1-antitrypsin deficiency (AATD) is a rare hereditary condition characterised by low plasma levels of alpha1-antitrypsin (AAT), a serine protease inhibitor synthesised and secreted mainly by hepatocytes, of which the primary role is to protect the lung parenchyma from proteolytic enzymes such as neutrophil elastase (NE) and proteinase 3. The disease is caused by mutations in the *SERPINA1* gene. Clinical manifestations include pulmonary emphysema; liver cirrhosis; and, in rare cases, necrotising panniculitis and antineutrophil cytoplasmic antibody (C-ANCA)-positive vasculitis [1]. Current American Thoracic Society (ATS) and European Respiratory Society (ERS) guidelines recommend testing plasma AAT levels in individuals with Chronic Obstructive Pulmonary Disease (COPD), unexplained chronic liver disease, bronchiectasis, panniculitis, or granulomatosis with polyangiitis, along with the parents, siblings, and children of individuals with a mutated AAT allele. Despite these recommendations, AATD is highly underdiagnosed [2]. AATD is one of the most common inherited disorders [3], with a prevalence of 1–5/10,000. It is estimated that about 3.4 million individuals worldwide have deficient allele genotypes that lead to AAT deficiency [4]; nevertheless, more than 90% of affected subjects remain underdiagnosed [3,5]. A diagnostic delay is observed with an average interval of 8.3 years between onset of pulmonary symptoms and diagnosis and consultations with several clinicians before diagnosis [5] (an average of 2.7 physicians), leading to irreversible lung function impairment, which could be delayed by establishing clinical controls and healthy lifestyle habits in childhood or early stages of the disease.

Primary ciliary dyskinesia (PCD) is a rare hereditary disorder with autosomal recessive inheritance, characterised by altered or absent ciliary movement, which generates mucociliary clearance deficit [6]. Prevalence is estimated at around 1/10,000 live births [7]. Clinical manifestations include neonatal respiratory distress of unknown cause; presence of *situs inversus*, ventriculomegaly, constant rhinorrhoea, chronic productive cough, or bronchiectasis of unknown cause; immobile sperm in adult males; and recurrent ectopic pregnancies in women [8,9]. Symptoms are early and recurrent from the first years of life, but as they are linked to respiratory infections, frequent in childhood, it is easy to underestimate the existence of this disease. In addition, diagnostic tests involve complex studies of ciliary motility, electron microscopy, and genetic tests, which are inaccessible in many centres and yield difficult-to-interpret results.

Prompt diagnosis is important in both diseases because early treatment helps slow progression. Therefore, the aim of the present work was to evaluate whether the under- and delayed diagnosis observed was due to a lack of knowledge of these diseases by medical doctors who manage these patients at the paediatric age (primary care paediatricians and paediatric specialists in pulmonology and gastroenterology). To this end, a series of surveys assessing physicians’ knowledge of these rare diseases were prepared. Medical students were also included in the survey to ascertain whether the problem is due to insufficient medical school training (since education in medical schools is generalist and based primarily on knowledge of highly prevalent diseases) or to the impossibility of gaining experience in clinical practice because of the rarity of these pathologies together with limited training recycling in this area.

## 2. Materials and Methods

### 2.1. Study Design, Setting, and Participants

An analytical, observational, and cross-sectional study was carried out using anonymous surveys at the Paediatric Pulmonology Unit of the Hospital Clínico Universitario Valencia (HCUV) from January 2015 to January 2017.

All members of the Valencian Society of Paediatrics (SVP) (1241 members); Spanish Society of Paediatric Pulmonology (SENP) (275); and Spanish Paediatric Gastroenterology, Hepathology and Nutrition Society (SEGHNP) (400) were invited to participate in the study, as were all students enrolled in the final year of the Faculty of Medicine of the University of Valencia (271 students). The Supplementary file shows the specific questionnaires developed for AATD and for PCD, respectively. Both questionnaires were validated by four experts in both pathologies (see Supplementary file for further explanations on validation). After project approval from the Boards of Directors of the Scientific Societies involved and by the Dean of Valencia School of Medicine, a letter of invitation was sent by e-mail to all members of the institutions and to final-year medical school students requesting their participation.

A separate online data-collection questionnaire was used for each of the two diseases studied, using a web platform called *Typeform*, which permits centralised data collection. Members of the SEGHNP answered a 12-question AATD survey (Supplementary file), while members of the SVP and SENP and medical students completed a test on both diseases, which consisted of 21 questions (Supplementary file). The surveys included four questions on professional profile (years of clinical experience, type and location of work centre, and paediatric medical speciality) and on the number of children diagnosed. Questions on self-assessment and a test of diagnostic and therapeutic competence in both diseases were also included. Overall, maximum scores were set at 7 points for the AATD and at 17 for the PCD tests. Both surveys took place in parallel. Correct answers are shown in Supplementary file.

Data were collected on an individual basis. Confidentiality was maintained in all surveys in accordance with Spanish personal data protection laws. After requesting approval from the societies’ boards of directors and the Dean of the University of Valencia medical school, an invitation letter was sent by e-mail (through the medical societies and the medical school, which have legitimate access to such mailings) to the members of the aforementioned societies and to the students, requesting their participation in the study. No personal data were collected in the questionnaires. Answers collected from the online form were automatically stored in a database and then converted to a numerical scale for processing.

### 2.2. Statistical Analysis

Data recorded on the *Typeform* web platform were exported to the statistical software “IBM SPSS Statistics for Windows, Version 20.0” (IBM Corp, Armonk, NY, USA) for further analysis. A descriptive study of the sample was conducted, separating the results for the two diseases. Qualitative variables are shown as frequency and percentages, while for quantitative variables, data are shown as mean and standard deviation (SD). Assessment of normality was performed using the D’Agostino–Pearson normality test. Comparison between clinical specialities was performed using chi-square or Fisher test for qualitative variables and by ANOVA or by Student’s t-test (normal distribution) or Mann–Whitney U test (non-normal distribution) for quantitative variables. Differences were considered statistically significant when *p* was <0.05.

## 3. Results

### 3.1. AATD Knowledge

The demographic characteristics and professional experience of the individuals participating in the AATD/PCD knowledge survey are shown in Table 1; 618 surveys were completed on AATD with the following distribution by groups: (i) 193 General Paediatricians (GP); (ii) 123 Paediatric Pneumologists (PP); (iii) 166 Paediatric Gastroenterologists (PG); and iv) 136 Medical School students (MS).

Regarding years of clinical practice, about one-quarter of GP (25.3%) and PG (28.3) and 7.3% of PP had less than five years’ clinical experience. Characteristics of respondents with 5–15 years of clinical practice were more homogeneous between groups, showing similar percentages in GP (35.2%) and PG (32.0%) with a higher percentage of PP (43.1%). Similar results were observed in the group with more than 15 years of clinical practice, in which GP (39.3%) and PG (39.7%) showed similar percentages, with a higher percentage of PP (49.6%).

Regarding the type of healthcare practice, 57.5% of the GPs who completed the survey worked in primary care, while around 64.2% of the PPs and 63.2% of PGs worked in public tertiary hospitals. Paediatric pneumologists were considered the paediatric specialists best prepared to diagnose AATD by all the groups that participated in the survey (selected by 59% of paediatricians, 86.1% of gastroenterologists, 93.5% of pneumologists, and 35.2% of medical students). Medical doctors were notably unaware of the existence both of reference units for AATD (unknown to 70% of PPs and 77.1% of PGs) and of the Spanish Register of AATD (REDAAT) (unknown to 67.8% of those surveyed).

In the self-evaluation section, 61.7% of MSs and 45.6% of GPs admitted to having a very basic knowledge of the disease, while the majority of paediatric specialists (78.8% of pulmonologists and 62% of gastroenterologists) positively self-evaluated on diagnostic procedures. The professionals surveyed reported screening serum levels of AAT in children with hepatopathy or transaminase elevation (40.9%), repeat pneumonias (39.9%), or bronchiectasis (34.1%).

Regarding level of expertise on clinical manifestations of the disease [1] (Table 2), 39.8% of PPs and 31.9% of PGs got two or three answers correct, compared to 16% of GPs and 2.1% of MSs. Responses regarding the most severe AATD phenotype [1] were quite satisfactory: 93.5% of PPs, 86.1% of PGs, and 59.1% of GPs answered correctly, whereas 35.3% of MSs got it right. The results are definitively unbalanced in the third question about treatment/management of AATD in children [1,10,11,12,13,14,15]. Only 25.9% of GPs, 25.2% of PPs, and 21.7% of PGs choose the three correct options. This percentage was reduced to 14.7% in MSs (Table 2).

**Figure 1:**
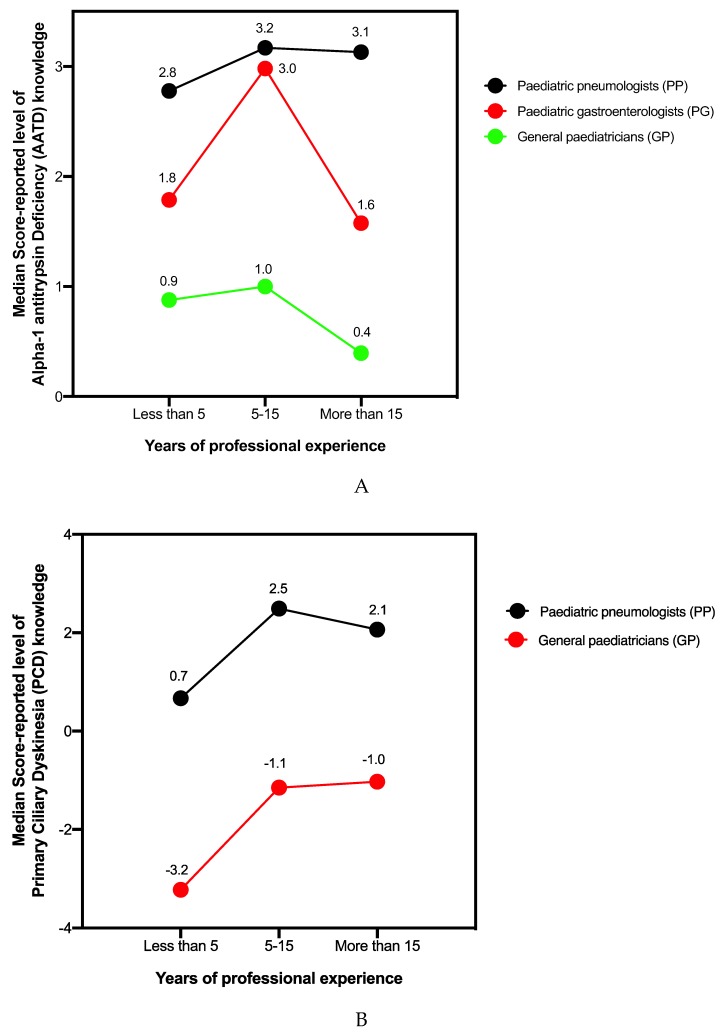
Alpha-1 Antitrypsin Deficiency (AATD) and Primary Ciliary Dyskinesia (PCD) knowledge in paediatric specialists in relation to years of professional experience: No group reached the minimum score required to make an early diagnosis and to adopt the appropriate measurements for correct management of patients with AATD (**A**) or PCD (**B**) regardless of years of professional experience. Maximum scores were 7 points for the AATD and 17 for the PCD tests.

When analysing the average score of the four groups surveyed, the highest score corresponds to the group PP (3.12 points) which, nonetheless, failed to reach the theoretical 50% set as the minimum level to “pass”, a fact that reveals the respondents’ low AATD knowledge level (Figure 1A).

A statistically significant relationship between professional profile and AATD test score (*p* < 0.001) was observed, with PPs achieving a significantly higher average than the rest. Also significant is the relationship between years of experience and score achieved (*p* = 0.045), with the particularity that the group of professionals with intermediate experience (between 5 and 15 years) were shown to be the most up-to-date on AATD, with higher average scores than those with greater or lesser experience. No significant relationship was found between subjective perception of competence by the different groups analysed (self-evaluation) and results obtained in the test (*p* = 0.139) or between degree of awareness the disease and type of healthcare practice, with the exception of the PP group (*p* = 0.008).

Finally, experience of treating patients with AATD correlated significantly with the average score obtained in the test on this pathology in PPs and PGs (*p* = 0.029 and *p* < 0.001, respectively), in contrast with the GP group (*p* = 0.329).

### 3.2. PCD Knowledge

Of the 457 respondents to the PCD survey, 190 were GPs, 123 were PPs, and 135 were MSs (Table 1). Professional experience was also very disparate, being more homogeneous among GPs (<5 years of experience: 25.78%; 5–15 years: 35.78%; and >15 years: 38.42%) and longer among PPs (43% 5 to 15 years and 49.6% over 15 years). As for the type of healthcare practice, 57.3% of GPs worked in health centres and 64.2% of PPs worked in tertiary public hospitals.

The professionals considered most suitable for diagnosing PCD were also paediatric specialists; specifically, the paediatric pulmonologist was chosen by 243 of those surveyed.

In the study, 66.6% of PPs stated that they knew where to refer this type of patient for more specialised diagnosis and/or follow-up. However, 78.9% of GPs and 91.1% of MSs were unaware of the existence of referral units for this disease. These data are inconsistent with their own self-evaluation, since around 50% of the three populations analysed (49.5% of GPs, 58.5% of PPs, and 54.4% of MSs) claimed to know PCD.

Based on a total score of 0–7, more than one-half of the GPs (52.6%) and MSs (77.7%) obtained a score of less than 50% (Table 3), nowhere near the results of PPs (49.59% correctly identified all early symptoms). However, this apparently excellent result is overshadowed by a not inconsiderable percentage (38.2%) that considered some late clinical manifestations such as bronchiectasis to be early ones or that incorrectly identified others as characteristic symptoms of PCD (e.g., recurrent bronchial obstructive crisis).

In the section on diagnostic options (Table 3), with a maximum score of 10 points, 27.36% and 26% of GPs and MSs, respectively, obtained more than 50% of correct options, whereas 43.9% of PPs obtained five or more correct answers [16,17,18,19,20,21,22].

After penalising respondents for incorrectly selected options, the average scores obtained by GPs, PPs, and MSs were −1.64, 2.15, and −2.47 points, respectively, out of a total 17 possible points, denoting very low overall PCD knowledge (Figure 1B).

Not unexpectedly, the results are closely related to the respondent’s professional profile (*p* < 0.001), with PPs showing the greatest knowledge of the disease, despite not reaching the minimum 8.5 points (50% of the maximum score) required to pass. The overall test score does not correlate with years of professional experience (*p* = 0.778), self-assessment (*p* = 0.687), or work centre (*p* = 0.132). Only the GP group showed a significant correlation with having diagnosed patients with PCD (*p* = 0.023), absent in PPs (*p* = 0.163).

Correct diagnostic options in childhood:

- Not definitive for PCD diagnosis: audiometry [17], sperm motility [18], saccharin test [17].
- Partially diagnostic: chest and/or sinus x-ray [16,19], pulmonary CT, spirometry and/or plethysmography and/or diffusion test [20].
Diagnostic screening tests: nasal nitric oxide [16], mucociliary clearance test with radioisotopes [21].
Determining diagnostic tests: ciliary motility (pattern and speed) and ciliary ultrastructure [16,21,23].

## 4. Discussion

In rare diseases, early diagnosis is key and represents one of the main problems faced by patients and their families. Underdiagnosis and delayed diagnosis are relatively constant, probably due to the insufficient academic training on this subject in medical schools, where teaching focuses mainly on the most prevalent pathologies.

Identifying the cause of a problem is the first step towards a possible solution. Various studies have shown that lack of knowledge of rare diseases by medical doctors is a cause of under- and delayed diagnosis [5,23,24]. However, despite the fact that, in a high percentage of cases, paediatricians are at the front line in attending this type of patient/pathology, no study so far has evaluated their knowledge level in these areas, hence our proposition focusing on two of the most prevalent rare respiratory diseases, AATD and PCD, and also including students in their final year at the School of Medicine of the University of Valencia (Spain) in order to assess the level of academic training received.

We collected a total of 1081 surveys, 624 on AATD and 457 on PCD, widely surpassing the number of participants in other previous studies and focusing on previously unstudied populations: paediatricians, paediatric specialists, and final-year degree students.

The results of the two surveys showed that both MSs and GPs are unfamiliar with most of the signs or symptoms on which diagnostic suspicion of both diseases is based and are also unaware of the steps to be taken to arrive at a definitive diagnosis or to enter the relevant healthcare circuit.

As expected, familiarity with these processes is higher among PP who also have greater work experience (49.6% practicing for more than 15 years); however, the difference cannot be explained by this circumstance alone, since the number of cases diagnosed or treated by each professional is very low, regardless of their years of professional service.

No group reached the minimum score required to be able to make an early diagnosis and to adopt the appropriate measures for correct management of patients with AATD or PCD. This low formation contradicts the self-evaluations of respondents who overestimated their knowledge of both diseases; this further aggravates the problem because unawareness of shortcomings precludes learning. In the case of AATD, 45.6% of GPs and 61.7% of MS claimed to know the disease, while 78.8% of PPs and 62% of PGs believed they knew the diagnostic procedure. In PCD, half of those surveyed (49.5% of GPs, 54.4% of MSs, and 58.5%, of PPs) considered themselves competent in the disease.

In light of the above, it is particularly striking that half of the GP surveyed claimed to have attended patients with AATD (50.6%) and/or PCD (41.6%) when the low prevalence of these rare diseases limits this possibility. In the case of PCD, GP would not be the ones to diagnose these patients due to the specialised and complex techniques required for diagnosis only available at two centres in Spain (Valencia and Barcelona). This also raises doubt as to whether many PCD cases are correctly classified by GPs. This is not an issue among PPs because their specialty requires a greater degree of information/training on PCD and they do not rely exclusively on having diagnosed patients.

Analysing the responses to questions 9 and 16, PPs are the physicians considered most suitable for diagnosing both clinical entities, which is fully in line with ATS/ERS recommendations on AATD [2,25] and PCD [17], which advocate arriving at an early diagnosis during childhood.

Paediatricians’ lack of awareness about reference units for either disease is also noteworthy. This could be due in the case of AATD to the scarce/null expression of the disease during paediatric age but not in PCD, since one of the two Spanish referral units is in Valencia, where this study was carried out. It is even more surprising considering that 425 of the 457 surveyed viewed PCD as a complex disease requiring diagnostic confirmation and management in specialised centres.

Turning to screening, since the initial AATD diagnosis is performed by measuring AAT plasma levels, it was interesting to know in which cases the participating paediatricians requested this test, a question previously posed only to doctors attending adult populations [5,23,24]. It is accepted that AATD should always be ruled out in the blood relatives of patients with this diagnosis even if they are asymptomatic and in cases of neonatal jaundice or hypertransaminasemia, regardless of age, especially if there is a previous history of jaundice or liver disease. The association between AATD and asthma is controversial, with contradictory publications regarding the presence of Z alleles in asthma patients. The ATS–ERS-published consensus [2,25] recommends testing in adults with nonatopic asthma and in individuals with unexplained liver disease, including neonates and children. In our study, more than 50% of the physicians surveyed claim to request AAT levels in the clinical circumstances mentioned (Table 3) and almost 25% of PPs routinely include it when requesting any other blood test from their patients. If so, one would expect that early diagnosis of this disease would be less uncommon than it has been to date.

Contrary to expectations, working in tertiary hospitals does not seem to improve awareness of AATD and PCD. As far as the latter is concerned, the fact that most Spanish hospitals are not equipped to carry out the complete diagnosis probably explains why professionals are not familiar with the disease.

Finally, medical students were the worst performers in all questions raised about both diseases. This highlights the limited information received on rare diseases during their university studies and the need to rectify this situation. On this note, it is worth mentioning that the first course on rare diseases in Spain has been included in the Valencia School of Medicine syllabus [26]. The main objective of this course is not that students know ALL rare diseases, which would not be realistic (since there are between 6000 and 8000 rare diseases), but to generate an academic and formative space which provides tools for students and future health professionals to cope with an orphan disease. More specifically, the aim is to familiarise the student with aspects such as what a rare disease is, how and when to suspect that a patient suffers from a rare disease, the main problems faced by rare disease patients, the problems of underdiagnosis and delayed diagnosis, where to find information once the patient has been diagnosed, the lack of specific treatments, the need to boost research on rare diseases, the importance of patient associations, etc. In other words, it is intended to fill the gap in specific training in this field by developing general competencies in the field of rare diseases without focusing on individual rare diseases. Importantly, rare diseases are an opportunity, as they serve as models for diseases of high prevalence. There are numerous examples. In particular, research on familial hypercholesterolemia has contributed to the development of statins, drugs used daily by millions of people worldwide to lower high cholesterol levels and to prevent the development of cardiovascular diseases. In our view, this undoubtedly commendable initiative should be adopted in other universities worldwide.

## 5. Conclusions

There are significant knowledge gaps regarding AATD and PCD among medical students and paediatricians—the physicians responsible for diagnosing these diseases early—and this shortcoming is not even recognised by the majority. Our results indicate that it is necessary to increase rare respiratory diseases training among all physicians responsible for suspecting and diagnosing them, which will allow early diagnosis and the setup of preventive measures and appropriate early-stage treatment.

## Figures and Tables

**Table 1:** Demographic characteristics and professional experience of the individuals participating in the alpha-1 antitrypsin deficiency/primary ciliary dyskinesia (AATD/PCD) knowledge study.

Demographic Data											Professional Experience			
		Years of Clinical Practice			Healthcare Practice Type						AATD/PCD Patient Management		Awareness of Reference Units	
Groups	Number of Surveys Completed	<5	5–15	>15	Primary Care (Clinic)	Private Hospital	Public Tertiary Hospital	District Hospital	Private Practice	Public Secondary Hospital	Yes	No	Yes	No
General paediatricians (GP)	**A:** 193	25.3%	35.2%	39.3%	57.5%	2.6%	19.7%	17.1%	2.1%	1.0%	48.7%	51.3%	14.5%	85.5%
	**B:** 190	25.7%	35.7%	38.4%	57.3%	2.6%	19.4%	17.3%	2.1%	1.3%	46.8%	52.1%	19.7%	80.3%
Paediatric pneumologists (PP)	**A:** 123	7.3%	43.1%	49.6%	--	3.2%	64.2%	24.4%	2.4%	5.7%	52.0%	48.0%	30.0%	70.0%
	**B:** 123	7.4%	43.0%	49.6%	--	3.2%	64.2%	24.3%	2.4%	5.7%	47.1%	52.9%	66.6%	33.3%
Paediatric gastroenterologists (PG)	**A:** 166	28.3%	32.0%	39.7%	--	8.4%	63.2%	26.5%	1.2%	0.6%	66.9%	33.1%	22.9%	77.1%
Medical students (MS)	**A:** 136	--	--	--	--	--	--	--	--	--	32.3%	67.7%	9.5%	90.4%
	**B:** 135	--	--	--	--	--	--	--	--	--	--	--	8.9%	91.1%

**A**: AATD knowledge survey. **B**: PCD knowledge survey.

**Table 2:** Correct answers to the alpha-1 antitrypsin deficiency (AATD) knowledge questionnaire.

Groups	Number of Correct Answers on the Clinical Manifestations of the Disease				Correct Answers about the Phenotype of the Disease	Number of Correct Answers about the Treatment of the Disease			
	**0**	**1**	**2**	**3**		**0**	**1**	**2**	**3**
**General paediatricians (GP)**	44.5%	38.5%	15.5%	1.50%	59.1%	16.1%	24.9%	33.2%	25.8%
**Paediatric pneumologists (PP)**	12.2%	47.9%	37.4%	2.4%	93.5%	9.8%	26.8%	38.2%	25.2%
**Paediatric gastroenterologists (PG)**	18.6%	49.4%	30.1%	1.8%	86.1%	21.7%	25.3%	31.3%	21.7%
**Medical students (MS)**	77.9%	19.8%	1.4%	0.7%	35.3%	32.4%	23.5%	29.4%	14.7%

Correct symptoms [1]: neonatal jaundice; transaminases elevation; no expression. Correct phenotype [1]: most severe phenotype = ZZ. Correct treatment [1,10,11,12,13,14,15]: balanced diet rich in antioxidants; early prevention/control of respiratory infections and bronchial inflammation; avoiding active/passive smoking and environmental pollution.

**Table 3:** Correct answers to primary ciliary dyskinesia (PCD) knowledge questionnaire.

Groups	Correct clinical Manifestations								Correct Diagnostic Options										
	0	1	2	3	4	5	6	7	0	1	2	3	4	5	6	7	8	9	10
**General paediatricians (GP)**	2.6%	13.1%	21.0%	15.7%	14.2%	13.6%	11.5%	7.8%	0.5%	4.7%	20.0%	28.4%	18.9%	15.7%	10.0%	1.05%	--	0.5%	--
**Paediatric pneumologists (PP)**	--	1.6%	6.5%	8.1%	4.8%	13.0%	16.2%	49.5%	--	1.6%	8.1%	21.9%	24.3%	25.5%	8.9%	8.1%	3.5%	--	--
**Medical students (MS)**	17.0%	22.9%	25.1%	12.5%	8.8%	9.6%	1.4%	2.2%	4.4%	8.1%	18.5%	19.2%	23.7%	18.5%	5.1%	1.4%	0.7%	--	--

Correct clinical manifestations: onset of symptoms in neonatal period, *situs inversus*; neonatal respiratory distress with no apparent cause; constant/persistent rhinorrhoea; recurrent or chronic moist cough; recurrent otitis; repeat pneumonia.

## Supplementary Materials

The following are available online at https://www.mdpi.com/2077-0383/9/3/869/s1, Supplementary file: AATD Survey with Answers and PCD Survey with Answers and Validation Procedures.
