# Supplementary material for: Knowledge of Rare Respiratory Diseases among Paediatricians and Medical School Students
Source: J Clin Med. 2020 Mar 22;9(3):869. doi: 10.3390/jcm9030869 (PMC7141530; doi:10.3390/jcm9030869)
Supplement: Supplementary file 1 [file jcm-09-00869-s001.pdf]

ALFA-1 ANTITRYPSIN DEFICIENCY SURVEY

(Cross out or circle the correct answer)

Correct answers are labelled in yellow

1. How many years of professional experience do you have?

a) Less than 5
b) Between 5 and 15
c) More than 15

2. In which type of centre do you work?

a) Primary care
b) Private hospital
c) Public tertiary hospital
d) Regional hospital
e) Private clinic
f) Other- please specify:

3. What is your dedication?

a) General paediatrician
b) Paediatric pneumologist
c) Paediatric gastroenterologist
d) Medical student
e) Other- please specify:

4. Please, indicate in which province do you work?

5. Do you have, or have had, a case of AATD among your patients?

a) Yes
b) No

6. How much knowledge do you have on AATD?

a) I have no previous knowledge of this disease
b) I know it exists

c) I can identify the symptoms

d) I know the diagnostic procedure

7. What is the symptom, <u>in childhood</u>, that you consider the most representative of this disease? Several options may be chosen if necessary.

a) Asthmatic symptomatology

b) Repetitive pneumonia

c) Neonatal jaundice

d) High transaminases

e) No expression

f) I don't know

g) Other- please specify

8. What phenotype do you consider to confer the highest risk for AATD patients?

a) MS

b) MZ

c) MM

d) ZZ

e) SZ

f) Any of the above

9. Who do you think should diagnose AATD?

a) General Paediatrician

b) Pneumologists

c) Gastroenterologists

d) Paediatric Pneumologists

e) Paediatric Gastroenterologists

f) Any of the above

g) Other

10. In the case of a child diagnosed with AATD, what do you think would be the best management? Several options may be chosen if necessary.

a) Treatment with alpha-1 antitrypsin

b) Inhaled corticotherapy

c) Balanced diet rich in antioxidants

d) Liposoluble vitamins and liver protectors

e) Early prevention/control of respiratory infections and bronchial inflammation

f) Avoid active/passive smoking and environmental pollution.

g) None during infancy

h) There is no effective treatment for this disease

i) Other- please specify

11. When do you request an alpha 1 blood antitrypsin test? Several options can be selected

a) At least once, in all my patients, if I have to perform a blood test for any cause.
b) In children with liver disease or elevated transaminases
c) In children with asthma symptoms that are difficult to control
d) In children with repeated pneumonias
e) In children with bronchiectasis
f) In children of parents with AATD

12. Do you know any centre in Spain that specialises in the diagnosis and treatment of this disease?

a) Yes; please indicate here:

b) No

13. Are you aware of the existence of the " Spanish Registry of Patients with AATD (REDAAT)"?

a) Yes

b) No

ALPHA 1 ANTITRYPSIN DEFICIENCY and PRIMARY CILIARY DYSKINESIA SURVEY

(Cross out or circle the correct answers)

Note that correct answers are labelled in yellow

1. How many years of professional experience do you have?

a) Less than 5
b) Between 5 and 15
c) More than 15

2. In which type of centre do you work?

a) Primary care
b) Private Hospital
c) Tertiary public hospital
d) Regional Hospital
e) Private practice
f) Other- Please specify:

3. What is your dedication?

a) General paediatrician
b) Paediatric pneumologist
c) Paediatric gastroenterologist
d) Medical student
e) Other- Please specify

4. In which province do you work?

5. Do you have, or have ever had, a case of AATD among your patients?

a) Yes
b) No

6. How much knowledge do you have on AATD?

a) I have no previous knowledge
b) I know it exists
c) I can identify the symptoms
d) I know the diagnostic procedure

7. What is the symptom, in childhood, that you consider most representative of this disease? Several options may be chosen if necessary.

a) Asthmatic symptomatology
b) Repeat pneumonia
c) Neonatal jaundice

d) High transaminase
e) No expression
f) I don't know.
g) Other- Please specify:

8. What phenotype do you consider to confer the highest risk for AATD patients?

a) MS
b) MZ
c) MM
d) ZZ
e) SZ
f) Any of the above

9. Who do you think should diagnose AATD?

a) General paediatrician
b) Pneumologist
c) Gastroenterologist
d) Paediatric Pneumologist
e) Paediatric Gastroenterologist
f) Any of the above
g) Other- Please specify:

10. In the case of a child diagnosed with AATD, what do you think would be the best management? Several options may be chosen if necessary.

a) Treatment with alpha 1 antitrypsin
b) Inhaled corticotherapy
c) Balanced diet rich in antioxidants
d) Liposoluble vitamins and liver protectors
e) Early prevention/control of respiratory infections and bronchial inflammation
f) Avoid active/passive smoking and environmental pollution.
g) None during infancy
h) There is no effective treatment for this disease.
i) Other- Please specify

11. When do you request an alpha 1 blood antitrypsin test? Several options can be selected

a) At least once, in all my patients, if I have to perform a blood test for any cause.
b) In children with liver disease or elevated transaminases
c) In children with asthma symptoms that are difficult to control
d) In children with repeated pneumonias
e) In children with bronchiectasis
f) In children of parents with AATD

12. Do you know of any centre in Spain that specialises in the diagnosis and treatment of this disease?

Please indicate:

13. Are you aware of the existence of the "Registro Español de Pacientes con DAAT (REDAAT)" (Spanish Registry of Patients with AATD)?

a) Yes
b) No

14. Do you know what primary ciliary dyskinesia (PCD) is?

a) Yes
b) No

15. Have you diagnosed any cases of PCD?

a) Yes
b) No

16. Who do you think should diagnose PCD? Several options can be selected.

a) General Paediatrician
b) Paediatric pneumologist
c) Adult Pneumologist
d) Otolaryngologist
e) Any of the above
f) Other- Please specify:

17. At what age can the first PCD symptoms appear?

a) Neonatal period
b) Early Childhood
c) Second childhood
d) Adolescence
e) Adulthood

18. Which of the following signs/symptoms would induce you to establish early PCD diagnostic suspicion? Several options can be selected

a) Presence of *Situs inversus*
b) Neonatal respiratory distress with no apparent cause.

c) Severe bronchiolitis or torpid evolution.
d) Constant/persistent rhinorrhoea.
e) Recurrent or chronic moist cough.
f) Emaciation
g) Repeating broncho-obstructive crisis
h) Repeated otitis
i) Repeat pneumonia
j) Bronchiectasis

19. Do you think diagnostic confirmation is necessary or would clinical diagnosis be enough?

a) It is not necessary to confirm the diagnosis because there is no treatment or cure.
b) This is a complex disease that would require diagnostic confirmation in a reference centre and follow-up in a Paediatric Pneumology Unit.

20. What is the value of the following tests or examinations for early diagnosis of PCD?

a. Audiometry

- Determinant
- Little determinant
- No determinant at all
- It is not decisive for the definitive diagnosis but it can serve as a screening test.

b. Chest and/or breast x-ray

- Determinant
- Little determinant
- No determinant at all
- It is not decisive for the definitive diagnosis but it can serve as a screening test.

c. Pulmonary CT scan:

- Determinant
- Little determinant
- No determinant at all
- It is not decisive for the definitive diagnosis but it can serve as a screening test.

d. Spirometry and/or plethysmography and/or diffusion test

- Determinant

- Little determinant

- No determinant at all

- It is not decisive for the definitive diagnosis but it can serve as a screening test.

e. Determination of nasal nitric oxide

- Determinant

- Little determinant

- No determinant at all

- It is not decisive for the definitive diagnosis but it can serve as a screening test.

f. Saccharin test

- Determinant

- Little determinant

- No determinant at all in childhood

- It is not decisive for the definitive diagnosis but it can serve as a screening test.

g. Mucociliary clearance test with radioisotopes

- Determinant

- Little determinant

- No determinant at all

- It is not decisive for the definitive diagnosis but it can serve as a screening test.

h. Ciliary motility test

- Determinant

- Little determinant

- No determinant at all

- It is not decisive for the definitive diagnosis but it can serve as a screening test.

i. Ciliary Ultra-structure

- Determinant

- Little determinant

- No determinant at all

- It is not decisive for the definitive diagnosis but it can serve as a screening test.

j. Sperm motility analysis

- Determinant

- Little determinant

- No determinant at all in childhood

- It is not decisive for the definitive diagnosis but it can serve as a screening test.

21. Do you know of any centre in Spain that specialises in the diagnosis and treatment of PCD? Please indicate:

Validation procedures

The questionnaire was planned and written by all members of the research group. Once this was done we tried to answer individually the questions which gave us an indication of the time taken to complete the survey. After that, we pass on the survey to four colleagues (MDs) from the hospital that fulfilled the eligibility criteria for the study, so that they can answer as real responders. Their opinions gave us an indication of timing, wording and routing errors. We also gave the survey to other four colleagues (also MDs) that not fulfilled the eligibility criteria that pretended to be experts in the field. We asked them to be as obstructive as possible, asking challenging questions and providing difficult responses. Answers by these two groups helped us to find out what they understood by certain questions or why they responded as they did which allowed us to amend questions accordingly. Once the proposed changes were made, we asked four experienced colleagues (experts in pneumology, gastroenterology, otorhinolaryngology and internal medicine who had previously diagnosed and treated AATD and/or PCD patients) who were capable of determining ambiguities and other possible mistakes both in the questions and answers, to complete the questionnaire and discussed with them about what they understood for every question and the way that they responded to them. Final changes were made according to their recommendations.
